# Egyptian rousette bats maintain long-term protective immunity against Marburg virus infection despite diminished antibody levels

**DOI:** 10.1038/s41598-017-07824-2

**Published:** 2017-08-18

**Authors:** Amy J. Schuh, Brian R. Amman, Tara K. Sealy, Jessica R. Spengler, Stuart T. Nichol, Jonathan S. Towner

**Affiliations:** 1 Viral Special Pathogens Branch, Division of High-Consequence Pathogens and Pathology, Centers for Disease Control and Prevention, Atlanta, GA 30333 USA; 2 Department of Pathology, College of Veterinary Medicine, University of Georgia, Athens, Georgia 30602 USA

## Abstract

Although bats are natural reservoir hosts for numerous zoonotic viruses, little is known about the long-term dynamics of the host immune response following infection and how these viruses are maintained in nature. The Egyptian rousette bat (ERB) is a known reservoir host for Marburg virus (MARV). Following infection of ERBs with MARV, virus-specific IgG antibodies are induced but rapidly wane and by 3 months post-infection the bats are seronegative. To determine whether reinfection of ERBs plays a role in MARV maintenance, we challenge groups of ERBs that were “naturally” or experimentally infected with MARV 17–24 months prior. No bats in either group exhibit evidence of MARV replication or shedding and all bats develop virus-specific secondary immune responses. This study demonstrates that infection of ERBs with MARV induces long-term protective immunity against reinfection and indicates that other factors, such as host population dynamics, drive MARV maintenance in nature.

## Introduction

Bats (order *Chiroptera*) have been implicated as natural reservoir hosts for numerous zoonotic viruses including coronaviruses^1^, filoviruses^2^, lyssaviruses^3^ and paramyxoviruses^4, 5^. Although much insight on the natural history of virus infection in bats has been gained from experimental infections^3, 6–11^ and mathematical modeling^12–15^, with the exception of Nipah and rabies viruses^16, 17^, relatively little is known about the long-term dynamics of the host immune response following primary virus infection. Of particular interest is whether or not long-term protective immunity is established following virus infection. This gap in knowledge can be attributed to: 1) the difficulties associated with obtaining serial biological samples from individual bats comprising large colonial, interconnected populations and 2) the uncertainty of whether short-term experimental models of virus infection in bats recapitulate natural bat-virus infection dynamics. Nonetheless, obtaining empirical data on the long-term dynamics of the bat immune response following virus infection is critical to understanding how bat-borne viruses are maintained in nature and identifying factors leading to virus spillover into humans.

The marburgviruses (family *Filoviridae*, genus *Marburgvirus*, Marburg virus (MARV) and Ravn virus (RAVV)) cause outbreaks of hemorrhagic disease in sub-Saharan Africa characterized by human-to-human transmission and high case fatality ratios^18^. The cave-roosting Egyptian rousette bat (ERB; *Rousettus aegyptiacus*) has been identified as a natural reservoir host for the marburgviruses and a source of virus emergence in humans^2, 19, 20^. A longitudinal ecological study of marburgvirus infection in large ERB populations at Python Cave and Kitaka Mine, Uganda revealed an age-associated cyclical pattern of virus infection in which pups (0.0% PCR prevalence) are seemingly protected from virus infection through maternal antibodies until becoming independent, young juveniles at roughly 3 months of age (2.7% PCR prevalence, 4.1% seroprevalence)^19^. Acute virus infection levels increase in the juvenile population, peaking at 6 months of age (12.4% PCR prevalence, 14.8% seroprevalence), coincidental with the timing of the biannual birthing seasons. Juveniles enter the adult population at 7–8 months of age; a population that experiences year-round, consistent levels of virus infection (2.4% PCR prevalence, 21.5% seroprevalence). A susceptible-exposed-infectious-resistant (SEIR) mathematical model of marburgvirus transmission in a closed ERB population of 40,000 individuals with a twice-yearly birth pulse and a 21-day latent period predicted a prevalence of active infection (<2.0%) comparable to that observed in the Python Cave ecological study (2.5%), but predicted a seroprevalence (100.0%) remarkably higher than that which was observed in adults^14^. This discrepancy is likely due to waning marburgvirus antibody levels that may or may not be indicative of diminished protective immunity^14^.

Serological data gathered from experimental studies of MARV infection in captive ERBs^8–11^ corroborate marburgvirus seroprevalence predictions generated by the SEIR model of marburgvirus transmission in ERBs^14^. Shortly after experimental inoculation of ERBs with MARV, the virus can be detected in the blood from 1-16 days post infection (DPI; 100% of bats for a mean duration of 6.0 d)^11^, the oral mucosa from 5–19 DPI (91.7% of bats for a mean duration of 4.6 d)^11^, multiple tissues from 2–12 DPI^8–10^ and the spleen up to 28 DPI (66.7% of bats at this time point)^8^. MARV IgG antibodies peak by 28 DPI and then rapidly decline^8–11^, falling below the threshold of seropositivity by 3 months post infection (MPI)^11^. Short-term protective immunity against viral replication and shedding has been demonstrated in seropositive ERBs challenged with homologous virus 48 days following experimental inoculation with a moderately high dose of MARV^9^. However, whether natural MARV infection generates long-term immunity sufficient to fully protect seronegative ERBs from viral reinfection, replication and shedding remains unclear.

We previously demonstrated horizontal MARV transmission between experimentally infected and naïve contact ERBs^11^. In this study, we assess whether MARV infection confers long-term protective immunity against reinfection, replication and shedding by challenging groups of ERBs that had been experimentally or “naturally” infected 17–24 months prior during the previous transmission study with homologous virus. Following challenge, evidence of MARV replication in the blood and viral shedding from the oral mucosa is monitored for 14 days, MARV IgG antibody responses are monitored for 21 days and tissues obtained at necropsy at 21 days are tested for the presence of MARV RNA. Herein, we show that no bats in either group exhibit evidence of MARV replication or shedding. Further, all bats develop virus-specific secondary immune responses, demonstrating that infection of ERBs with MARV induces long-term, and likely lifelong, protective immunity against reinfection.

## Results

### Bat groups and expectations

Group 1 was comprised of 5 bats that were experimentally infected with MARV 24 months previously, group 2 was comprised of 5 bats that were “naturally” infected through contact with the experimentally infected bats 17–18 months previously and group 3 was comprised of 2 negative control bats (Table 1). Following challenge of groups 1 and 2 with homologous MARV, we expected bats lacking protective immunity to exhibit viremias and viral RNA shedding from the oral mucosa prior to 14 days post challenge (DPC), viral replication in the tissues at necropsy at 21 DPC and a delayed virus-specific IgG immune response not detectable until 14 DPC. While bats possessing protective immunity against MARV reinfection were not expected to exhibit viremias, viral shedding from the oral mucosa or virus replication in the tissues, they were expected to attain robust virus-specific IgG antibody responses by 7 DPC.

**Table 1:** Details of the bats according to group.

**Group**	**Identification number**	**Sex**	**MARV RNA positive** ^**a**^ **(relative to time post primary infection of group 1 bats)**	MARV IgG antibody positive* (relative to time post primary infection of group 1 bats)
1	214605	M	2–17 d	14–56 d
	550417	F	2–16 d	14–56 d
	685734	M	3–14 d	14–56 d
	685891	F	5–18 d	14–56 d
	686146	M	3–8 d	14–42 d
2	684822	F		7–8 m
	684904	M	7 m	8 m
	720802	M		7 m
	721442	F		7–8 m
	726397	F		7–8 m
3	725904	M		
	726415	F		

^a^Blood, oral, rectal and/or urine specimens. *Group 2 bats seroconverted at the indicated time due to exposure to group 1 bats.

### No evidence of MARV replication or shedding

Consistent with the existence of long-term protective immunity against MARV reinfection, replication and shedding, none of the bats in groups 1 and 2 developed detectable viremias or shed MARV RNA in their oral secretions throughout the 14-day specimen collection period. All tissues, including the axillary lymph node, gonad, liver, salivary gland and spleen, collected from bats in groups 1 and 2 at necropsy on 21 DPC tested negative for MARV RNA. Throughout the study, MARV RNA was not detected in any of the samples collected from group 3 bats.

### Rapidly-attained immune response

**Figure 1:**
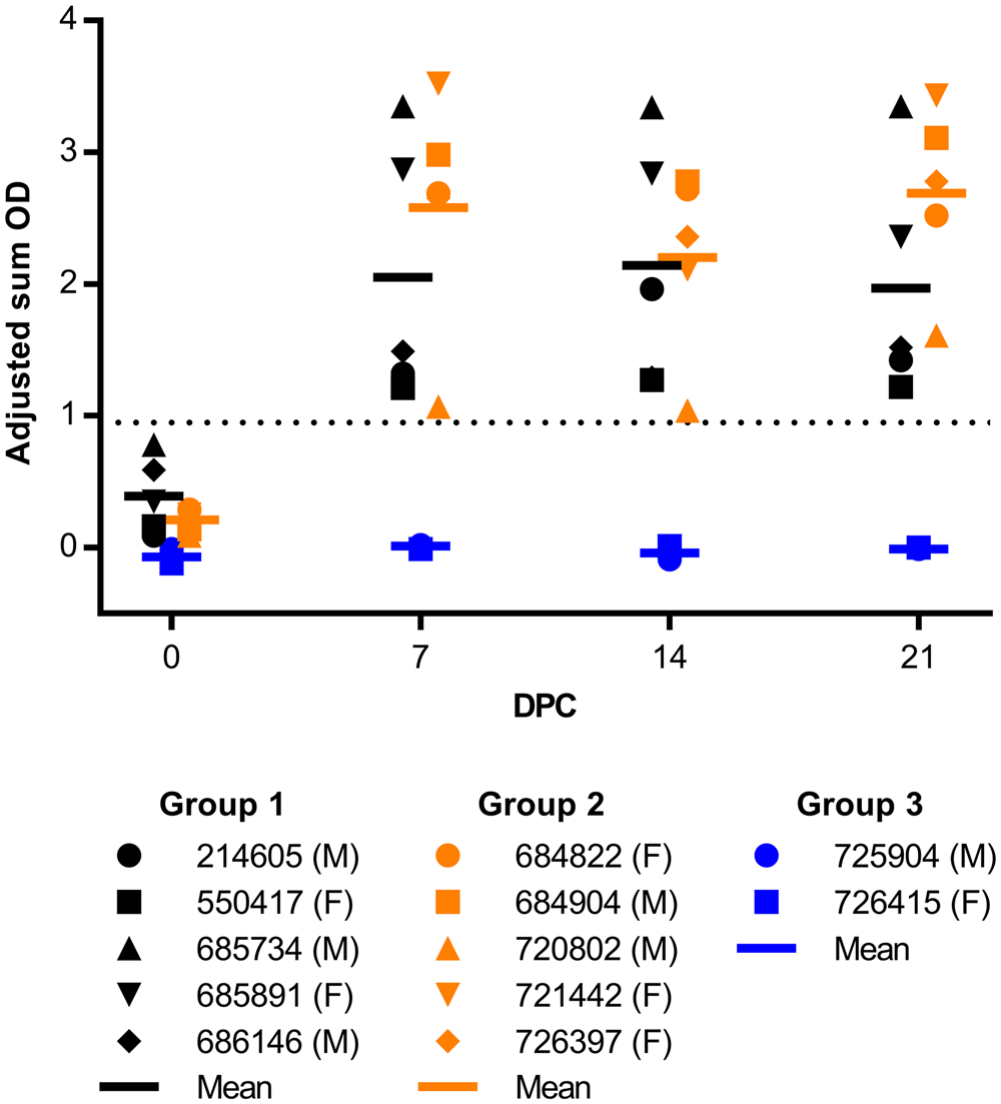
MARV IgG antibody responses of bats according to group. IgG antibodies were detected by ELISA with purified recombinant nucleoprotein of the Angola strain of MARV expressed in *Escherichia coli* from blood taken at 0, 7, 14 and 21 DPC. IgG antibody levels are expressed as adjusted sum OD values. The dotted line represents the threshold of the assay (MARV seropositive ≥ 0.95).

During the previous transmission study, MARV IgG (heavy and light chains) antibody levels for bats in groups 1 and 2 peaked and then rapidly declined falling below the threshold of seropositivity (adjusted sum optical density (OD) ≥ 0.95) within 3 months^11^. By the initiation of this study at 0 DPC, MARV IgG antibody levels remained below the threshold of seropositivity for bats in groups 1 (mean adjusted sum OD: 0.39, range: 0.09–0.78) and 2 (mean adjusted sum OD: 0.21, range: 0.09–0.29) (Fig. 1). By 7 DPC, bats in groups 1 (average adjusted sum OD: 2.05, range: 1.21–3.35) and 2 (average adjusted sum OD: 2.58, range: 1.07–3.52) had developed a robust MARV IgG antibody response. There were no statistically significant differences in MARV IgG antibody levels between groups 1 and 2 over time (F = 2.349, d.f._time*group_ = 1.688, d.f._error (time)_ = 13.504, two-tailed P = 0.138) or the sexes over time (F = 0.751, d.f._time*sex_ = 1.734, d.f._error (time)_ = 13.869, two-tailed P = 0.472). Group 3 bats tested uniformly MARV seronegative throughout the study.

## Discussion

The virus infection and immune response dynamics following reinfection with MARV are in stark contrast to those observed following primary infection, where the virus can be detected in the blood from 1–16 DPI infection (100% of bats for a mean duration of 6.0 d)^11^, the oral mucosa from 5–19 DPI (91.7% of bats for a mean duration of 4.6 d)^11^ and the spleen up to 28 DPI (66.7% of bats at this time point)^8^, and MARV IgG antibody levels are undetectable through 7 DPI, begin to rise at 9 DPI and peak between 14 and 28 DPI^8–11^. Prior to the initiation of this study, ERBs that were experimentally inoculated with MARV 24 months previously and those that were “naturally” infected through contact with infectious ERBs approximately 17–18 months previously were MARV seronegative^11^. Following subcutaneous challenge with a moderately high dose of MARV, virus was not detected in daily blood or oral swab specimens taken through 14 DPC and axillary lymph node, gonad, liver, salivary gland or spleen tissue taken at 21 DPC. A robust MARV IgG antibody response was observed at 7 DPC. The absence of MARV replication in the blood and spleen and virus shedding from the oral mucosa coupled with a rapidly attained, robust MARV IgG antibody response upon virus re-exposure is characteristic of a fully protective, secondary immune response.

Our data demonstrate that diminished MARV IgG antibody levels following primary infection are not associated with the loss of long-term protective immunity against virus reinfection, replication and shedding. This finding is in contrast to an experimental study on repeated rabies virus infection of big brown bats (*Eptesicus fuscus*), which found that primary infection followed by a transient virus-specific neutralizing antibody response was not able to protect all bats from reinfection and mortality upon challenge 6 months later^16^. Evidence for Nipah virus recrudescence was reported in an adult female large flying fox (*Pteropus vampyrus*) who was seropositive at study entry, seronegative 1 month later, shed infectious virus in the urine ~10 months later and then returned to being seropositive within 2 weeks^17^. Other studies have also observed rapidly waning virus-specific IgG or neutralizing antibodies following experimental infection of bats with Nipah^6, 7^, Hendra^6^ and Japanese encephalitis viruses^21^. Serological assays designed to detect virus-specific IgG or neutralizing antibodies in bats, particularly those with conservatively high seropositivity thresholds, likely underestimate the number of past infections as they are only able to detect those that have been infected recently (<3 months) or exposed to the virus more than once. This likely explains the discrepancy between observed and predicted seroprevalence in the SEIR model of marburgvirus transmission in ERBs^14^ and indicates that mathematical models of filovirus-natural host dynamics should continue to be parameterized using virus PCR prevalence data rather than seroprevalence data.

A previous study showed short-term protective immunity against MARV reinfection in seropositive ERBs challenged with homologous virus 48 days following experimental infection^9^. Here, we demonstrate long-term protective immunity against MARV reinfection in seronegative ERBs that were infected with homologous virus 17–24 months earlier. Annual survival probabilities estimated from demographic studies of frugivorous bats range from 0.43 (juvenile females) to 0.53 (juvenile males) for the black flying fox (*Pteropus alecto*)^22^, 0.43 (juveniles) to 0.83 (adults) for the straw-colored fruit bat (*Eidolon helvum*)^23^ and 0.47 (males) to 0.58 (females) for the common fruit bat (*Artibeus jamaicensis*)^24^. An exponential life table created from ~16,000 captures of ~9,000 *A. jamaicensis* estimated the average lifespan of this bat in the wild to be 1.6 years^24^. Based on these estimates, it is likely that the average lifespan of wild ERBs does not exceed 24 months and that primary MARV infection provides lifelong protective immunity against reinfection.

The results of this study indicate that reinfection of bats with MARV as a consequence of diminished protective immunity does not significantly contribute to virus maintenance in natural ERB populations. A combination of other factors, such as seasonal variation in host and environmental factors, large population sizes and metapopulation dynamics, likely drive MARV maintenance and prevent virus extinction. Seasonal variations in precipitation and temperature, as well as host aggregation, births, deaths and immunity, have been shown to play a major role in the persistence of many wildlife diseases^25^, including bat-borne viruses. Reduced mortality of big brown bats (*Eptesicus fuscus*) during winter hibernation has been associated with rabies virus maintenance^13^ and the birthing and lactation stages of the seasonal reproductive cycle in little red flying foxes (*Pteropus scapulatus*) have been linked to a higher risk of Hendra virus infection^26^. Marburgvirus prevalence data collected during the longitudinal ecological investigation of ERB colonies at Python Cave and Kitaka Mine, Uganda revealed that distinct pulses of virus infection in newly susceptible six-month old ERBs temporally coincided the biannual birthing season^19^, which occurs just before the peak of the rainy season^27^. The SEIR model of marburgvirus infection^14^ found that the virus was only able to persist when biannual breeding was incorporated into the model. Mathematical modeling of infectious agent-host dynamics indicate that pathogen persistence increases gradually with population size^28^ and measles virus, perhaps the most comprehensively modeled virus-host system, was not predicted to persist in isolated populations of less than 250,000 individuals^29, 30^. ERB colonies in tropical Africa where marburgviruses are found are large, often numbering over 100,000 individuals^19^, while ERB colonies in the Palearctic Region where marburgviruses have never been reported are considerably smaller, comprising no more than 1,500 individuals^31^. Accordingly, modeling predicted that marburgviruses were only able to persist in ERB populations > 20,000 individuals^14^. Extinction of infectious diseases in mobile populations is typically local as the agent can be reintroduced from spatially separated populations through the dispersal of infectious animals. Metapopulation dynamics have been shown to play a role in the persistence of measles virus in demographically heterogeneous human populations^32^, Hendra virus in flying foxes (*Pteropus spp*.) in Australia^15^ and Nipah virus in large flying foxes (*P. vampyrus*) and variable flying foxes (*Pteropus hypomelanus*) in Malaysia^33^. ERBs are patchily distributed throughout sub-Saharan Africa, with the limit of their geographic range extending north into southern Turkey and east into Pakistan^34^. Mark-recapture studies have documented movement patterns of ERBs ranging from local migration between roosts 35 km^35^ to 50 km^2, 19^ apart to long-distance migration up to 500 km^35^. Infectious marburgvirus has been exchanged through the sub-Saharan metapopulation network, as evidenced through the detection of homologous marburgvirus sequences from bats and humans throughout sub-Saharan Africa^19, 20^.

Although valuable information on the long-term dynamics of the ERB immune response to MARV was obtained from this study, the data should be interpreted in light of its limitations. This study used a small number of 2-year old, laboratory-bred bats that were contained in a controlled environment throughout the study. If more bats had been included in this study, we may have observed individual variations in response to MARV challenge. Unlike their wild counterparts, these experimental bats were not subjected to stressors including pregnancy, lactation, poor nutrition, predation, high ectoparasite burdens and infection with other pathogens. It is possible that exposure to natural stressors such as these could compromise the host immune system, resulting in increased susceptibility to reinfection with marburgviruses in nature. Lactation has been significantly correlated with coronavirus detection in *Myotis spp*. and *Pipistrellus spp*.^36^, pregnancy and lactation were significant risk factors for Hendra virus neutralizing antibodies in longitudinally-sampled spectacled flying foxes (*Pteropus conspicillatus*)^37^ and little red flying foxes (*P. scapulatus*)^26^, and nutritional stress was a significant risk factor for detectable Hendra virus neutralizing antibodies in longitudinally-sampled *P. scapulatus* ^26^.

In conclusion, this study provides empirical evidence that “natural” infection of ERBs with MARV confers long-term protective immunity against virus reinfection, replication and shedding. This indicates that MARV maintenance in the ERB population is likely driven by other factors such as influxes of susceptible juveniles provided by the twice-yearly birth pulse, large population sizes and metapopulation dynamics. Further research is needed to determine the specific mechanisms involved in MARV clearance and protective immunity.

## Methods

### Virus

The 371 bat strain of MARV (Uganda 371Bat2007, GenBank accession #: FJ750958) used in this study was isolated from pooled liver/spleen collected from an ERB in 2007 in southwestern Uganda^2^. Prior to use in this study, the virus was passaged twice in Vero-E6 cells (American Type Culture Collection (ATCC), Manassas, VA, USA; sex: female, authenticated by ATCC) in maintenance media (DMEM containing 2% Thermo Scientific HyClone fetal bovine serum (Fisher Scientific), 100 units/ml penicillin (Life Technologies), 100 µg/ml streptomycin (Life Technologies) and 2.50 µg/ml amphotericin B (Life Technologies)) at 37 °C in the presence of 5% CO_2_. The second passage stock virus (mycoplasma-free) was then titrated in the same cell line by tissue culture infectious dose 50 (TCID_50_) assay.

### Bats

The captive-born bats used in this study originated from the ERB breeding colony at the Centers for Disease Control and Prevention (CDC, Atlanta, GA, USA). This MARV-free colony was established from wild-caught ERBs imported from Uganda^8^.

All animal procedures were approved by the Institutional Animal Care and Use Committee (IACUC) at the CDC and performed according to the Guide for the Care and Use of Laboratory Animals (Committee for the Update of the Guide for the Care and Use of Laboratory Animals 2011). The CDC is an Association for Assessment and Accreditation of Laboratory Animal Care (AAALAC) fully accredited research facility.

Procedures conducted with MARV or MARV-infected bats were performed at the CDC under biosafety level 4 (BSL-4) laboratory conditions in compliance with Select Agent Regulations (Animal and Plant Health Inspection Service and Centers for Disease Control 2014). All bat cages were placed within bio-flow isolator units with HEPA-filtered inlet and exhaust air supplies (Duo-Flow Mobile Units, Lab Products Inc., Seaford, DE, USA).

All bats were group-housed in a climate controlled BSL-4 animal area, with a 12 h day/12 h night cycle. Bats were provided daily with their body mass in fresh fruit supplemented with protein/vitamin powder (Lubee Bat Conservancy, Gainesville, FL, USA) and received water *ad libitum*.

The number of bats used in this study was based on their previous MARV infection status (experimentally infected, “naturally infected or naïve negative control), as well as the amount of BSL-4 laboratory space required and the number of personnel needed to care for the bats over 24 months. The bat groups were sex-matched and no other methods of randomization were used throughout the study. Investigators were not blinded during the study.

### Experimental bat groups

A total of 12 healthy, adult ERBs (*Rousettus aegyptiacus*; 31 m of age; 6 males and 6 females; average weight of 164.0 g) were transferred from a previous 9-month long MARV transmission study that was initiated 24 months previously when the bats were 7 months of age (juveniles)^11^. The ERBs were divided into three groups (Table 1) based on their MARV infection history and sex. Group 1 was comprised of 5 bats (3 males, 2 females) that were subcutaneously inoculated at the beginning of the previous transmission study with 4 log_10_TCID_50_ of the 371 bat strain of MARV. Shortly after inoculation, MARV RNA/infectious virus was detected in blood, oral, rectal and/or urine specimens collected from these bats. MARV IgG antibodies in this group peaked between 14 and 28 DPI and then rapidly declined, with all bats becoming seronegative by 3 MPI. Group 2 was comprised of 5 bats (2 males, 3 females) that were “naturally” infected through contact with the MARV-experimentally infected bats (group 1), as evidenced through the detection of MARV RNA in blood or oral swab specimens at 7 MPI and/or MARV IgG antibodies between 7 and 8 MPI. Group 3 was comprised of 2 negative control bats (1 male, 1 female) that tested uniformly negative for MARV RNA and MARV IgG antibodies throughout the previous transmission study.

### Virus infection

At 0 DPC, bats in groups 1 and 2 were subcutaneously inoculated under isoflurane anesthesia with 4 log_10_TCID_50_ of the 371 bat strain of MARV prepared in 0.25 mL of sterile Dulbecco’s modified Eagle’s medium (DMEM; Life Technologies, Carlsbad, CA, USA) in the caudal abdominal region and bats in group 3 were inoculated in the same manner with 0.25 mL of sterile DMEM. Groups 1 and 2 were housed in separate cages maintained within a single bio-flow isolator unit, while group 3 was housed in a cage maintained in a separate bio-flow isolator unit.

### Specimen collection

Blood was taken daily from 0–14 DPC and at 21 DPC from the cephalic wing vein using a sterile lancet (C&A Scientific, Manassas, VA, USA). Blood was tested for the presence of MARV RNA by Q-RT-PCR through 14 DPC and MARV IgG antibody responses were monitored weekly through 21 DPC. The oral mucosa was sampled daily through 14 DPC using two polyester-tipped applicators (Fisher Scientific, Grand Island, NY, USA). One oral swab was tested for the presence of MARV RNA by Q-RT-PCR and the second swab was frozen in sterile media for attempted virus isolation of any MARV RNA positive swabs.

### Euthanasia and necropsy

At 21 DPC, the bats were euthanized by cardiac exsanguination under anesthesia followed by an overdose of isoflurane. At necropsy, the following tissues were collected: axillary lymph node, gonad, liver, salivary gland and spleen. One set of tissues was tested for the presence of MARV RNA by Q-RT-PCR and a second set were frozen for virus isolation attempts in the case that any tissues were MARV RNA positive.

### Nucleic Acid Extraction

Nucleic acid was extracted on the MagMAX Express-96 Deep Well Magnetic Particle Processor (Life Technologies) from gamma-irradiated-Rift Valley fever virus (RVFV)-spiked (RNA extraction positive control) blood and oral swab specimens using the MagMAX Pathogen RNA/DNA Kit (Life Technologies) and from tissues using the MagMax Total RNA Isolation Kit (Life Technologies).

### Q-RT-PCR

Reverse-transcribed MARV RNA, RVFV RNA and eukaryotic18S rRNA, was detected on the ABI 7500 Real-Time PCR System (Life Sciences, Grand Island, NY, USA) using the SuperScript III Platinum One-Step Q-RT-PCR Kit (Life Technologies) with amplification primers and reporter probes targeting the viral protein 40 (forward primer: GGA CCA CTG CTG GCC ATA TC, reverse primer: GAG AAC ATI TCG GCA GGA AG, probe 1: *56-FAM-*ATC CTA AAC*-ZEN-*AGG CTT GTC TTC TCT GGG ACT T*-3IABkFQ*, probe 2: *56-FAM-*ATC CTG AAT-ZEN-AAG CTC GTC TTC TCT GGG ACT T*-3IABkFQ*), large segment (forward primer: TGA AAA TTC CTG AGA CAC ATG G, reverse primer: ACT TCC TTG CAT CAT CTG ATG, probe: *FAM-*CAC AAG TCC ACA CAG GCC CCT TAC ATT G*-BHQ1*) and eukaryotic 18 S rRNA (Life Technologies) genes, respectively.

### Serology

ELISA plates were coated with 50 ng/well of purified recombinant MARV Angola nucleoprotein (NP) or Reston virus (RESTV) NP expressed in *Escherichia coli* (GenScript, Piscataway, NJ, USA) and then incubated overnight at 4 °C. After washing the plates, a 1:100 dilution of gamma-irradiated bat whole blood was added to the first well and 4-fold serial dilutions were performed through 1:6,400. Following a 1 h incubation at 37 °C, the plates were washed and bound antibody was detected using a 1:2,000 dilution of goat anti-bat IgG (Bethyl Laboratories, Montgomery, TX, USA, Cat#: A140-118P, Lot#: A140-118P-3). According to the manufacturer product datasheet, this antibody reacts specifically with IgG and with light chains common to other bat immunoglobulins, such as IgM. After incubation for 1 h at 37 °C, the plates were washed twice and the 2-Component ABTS Peroxidase System (KPL, Gaithersburg, MD, USA) was added. The substrate was incubated for 30 min at 37 °C prior to reading the plates on a microplate spectrophotometer at 410 nm. Adjusted sum optical density (OD) values were calculated by subtracting the ODs at each 4-fold dilution of wells coated with RESTV NP from their corresponding wells coated with MARV Angola NP. The average adjusted sum OD of duplicate runs was reported and the threshold for seropositivity was set at ≥ 0.95, as previously described^8, 11^.

### Data and statistical analyses

Figure 1 was created using GraphPad Prism 7.01 (GraphPad Software, La Jolla, CA, USA). Repeated measures ANOVA analyses were performed to determine if MARV IgG antibody levels differed significantly (two-tailed P < 0.05) between: 1) study group (group 1 = 5 bats; group 2 = 5 bats) over time and 2) sex (females = 5 bats; males = 5 bats) over time (SPSS Statistics 21, IBM Software, Armonk, NY, USA). Shapiro-Wilk tests (two-tailed P ≥ 0.05) concluded that the assumption of normality had been met for both datasets by verifying that MARV IgG levels were normally distributed at each level of: 1) study group and time, and 2) sex and time. Mauchly’s tests indicated that the assumption of sphericity had been violated for both datasets (1: χ^2^ = 12.479, d.f. = 5, two-tailed P = 0.030; 2: χ^2^ = 13.309, d.f. = 5, two-tailed P = 0.022), therefore degrees of freedom were corrected using Greenhouse-Geisser estimates of sphericity (1: ε = 0.563; 2: ε = 0.578).

### Data availability

The authors declare that all data supporting the findings of this study are available within the article or from the corresponding author upon request.
